# Functional characterisation of human synaptic genes expressed in the *Drosophila* brain

**DOI:** 10.1242/bio.016261

**Published:** 2016-04-11

**Authors:** Lysimachos Zografos, Joanne Tang, Franziska Hesse, Erich E. Wanker, Ka Wan Li, August B. Smit, R. Wayne Davies, J. Douglas Armstrong

**Affiliations:** 1 Brainwave-Discovery Ltd., Hugh Robson Building, 15 George Square, Edinburgh EH8 9XD, UK; 2 Parkure Ltd., Hugh Robson Building, 15 George Square, Edinburgh EH8 9XD, UK; 3 Neuroproteomics, Max Delbrück Center for Molecular Medicine, Robert-Rössle-Straße 10, 13125 Berlin, Germany; 4 Dept. Molecular and Cellular Neurobiology, Center for Neurogenomics and Cognitive Research, Neuroscience Campus Amsterdam, VU University Amsterdam, Amsterdam, De Boelelaan 1085, The Netherlands; 5 Institute of Adaptive and Neural Computation, School of Informatics, University of Edinburgh, Edinburgh EH8 9AB, UK

**Keywords:** *Drosophila melanogaster*, Fruit fly, Human, Synaptic, Synapse, Synaptic proteome, Synaptopathy, Mental illness, Characterisation, Courtship, Climbing

## Abstract

*Drosophila melanogaster* is an established and versatile model organism. Here we describe and make available a collection of transgenic *Drosophila* strains expressing human synaptic genes. The collection can be used to study and characterise human synaptic genes and their interactions and as controls for mutant studies. It was generated in a way that allows the easy addition of new strains, as well as their combination. In order to highlight the potential value of the collection for the characterisation of human synaptic genes we also use two assays, investigating any gain-of-function motor and/or cognitive phenotypes in the strains in this collection. Using these assays we show that among the strains made there are both types of gain-of-function phenotypes investigated. As an example, we focus on the three strains expressing human tyrosine protein kinase Fyn, the small GTPase Rap1a and human Arc, respectively. Of the three, the first shows a cognitive gain-of-function phenotype while the second a motor gain-of-function phenotype. By contrast, Arc, which has no *Drosophila* ortholog, shows no gain-of-function phenotype.

## Abstract (precis)

**Summary:** This report describes a resource collection of *Drosophila melanogaster* strains expressing human synaptic genes and methods that can be applied in order to investigate the genes' function.

## INTRODUCTION

Integrative analysis of the neuronal synapse proteome has uncovered an evolutionarily conserved signalling complex that underpins the cognitive capabilities of the brain (Pocklington et al., 2006; Emes et al., 2008; Fernández et al., 2009; Bayés et al., 2011) and is highly enriched with proteins linked to human neurological disorders including schizophrenia, depression, as well as neurodegenerative diseases, such as Huntington's, Alzheimer's and Parkinson's (Bayés et al., 2011; Grant, 2012). The synaptic proteome is highly dynamic (e.g. Dahlhaus et al., 2011), cell type specific (e.g. Hawrylycz et al., 2012) and intricately regulated (e.g. Coba et al., 2008, 2009). The high degree of complexity in the synaptic proteome combined with the highly polygenic nature of many brain disorders suggests systems biology approaches are likely to be the best route to unlock a new generation of neuroscience research and CNS drug development that society so urgently demands (Catalá-López et al., 2013). Yet these modelling type approaches also need fast, tractable *in vivo* models for validation.

More than 100 years after the discovery of the *white* gene in *Drosophila melanogaster*, the common fruit fly remains a key tool for the study of neuroscience and neurobiology. The fruit fly genome is well annotated and there is a vast genetic manipulation toolkit available. This allows interventions such as high throughput cloning (Bischof et al., 2013; Wang et al., 2012) and the precise insertion of transgenes in the genome (Groth et al., 2004; Venken and Bellen, 2007; Bischof et al., 2007; Markstein et al., 2008), the locally and temporally controlled expression of these genes using the Gal4/UAS system (Brand and Perrimon, 1993) and its derivatives such as Gal80^ts^, which in turn allow detailed manipulation of behaviour on the neuronal level (McGuire et al., 2003). In addition to the genetic toolkit there is an array of fruit fly behavioural responses that are well characterised and can be used to measure the effects of genetic interventions on a wide range of neuronal processes from locomotor activity and sensory perception through to cognition (Nichols et al., 2012; Vang et al., 2012). Many of the long list of ground-breaking discoveries in these fields (Bellen et al., 2010) relate to highly conserved components of neuronal synapses (Emes et al., 2008; Emes and Grant, 2012).

Here we describe the development of a resource collection of transgenic fruit fly strains that, along with a proposed framework of assays, can be used to study and characterise human synaptic genes and their interactions. The collection consists of strains of transgenic fruit flies expressing selected human synaptic genes, whereas the framework for the study and characterisation of these genes is based on a proposed set of phenotypic screening methods, in order to discover gain-of-function phenotypes from the expression of these genes. The screening methods assess a range of behavioural phenotypes from simple (locomotion) through to more complex (learning and memory). Although the coverage of the collection is far from comprehensive in terms of the synaptic proteome, the approach is easily replicable and extendable to include other variants or additional synaptic genes.

## RESULTS

### Humanised strain collection

We selected, on the basis of being reported in multiple mammalian synaptic proteomic studies (see Materials and Methods), 30 human synaptic proteins all linked to human or rodent behaviour, synaptic plasticity and disease. These include key members of the post-synaptic proteome functional, structural and regulatory components (e.g. Dlg4, Fyn, Arc, CamK2b) as well as genes with known associations with neurodegenerative disease (tau). A complete list of the genes selected and is given in Table 1.

**Table 1.**
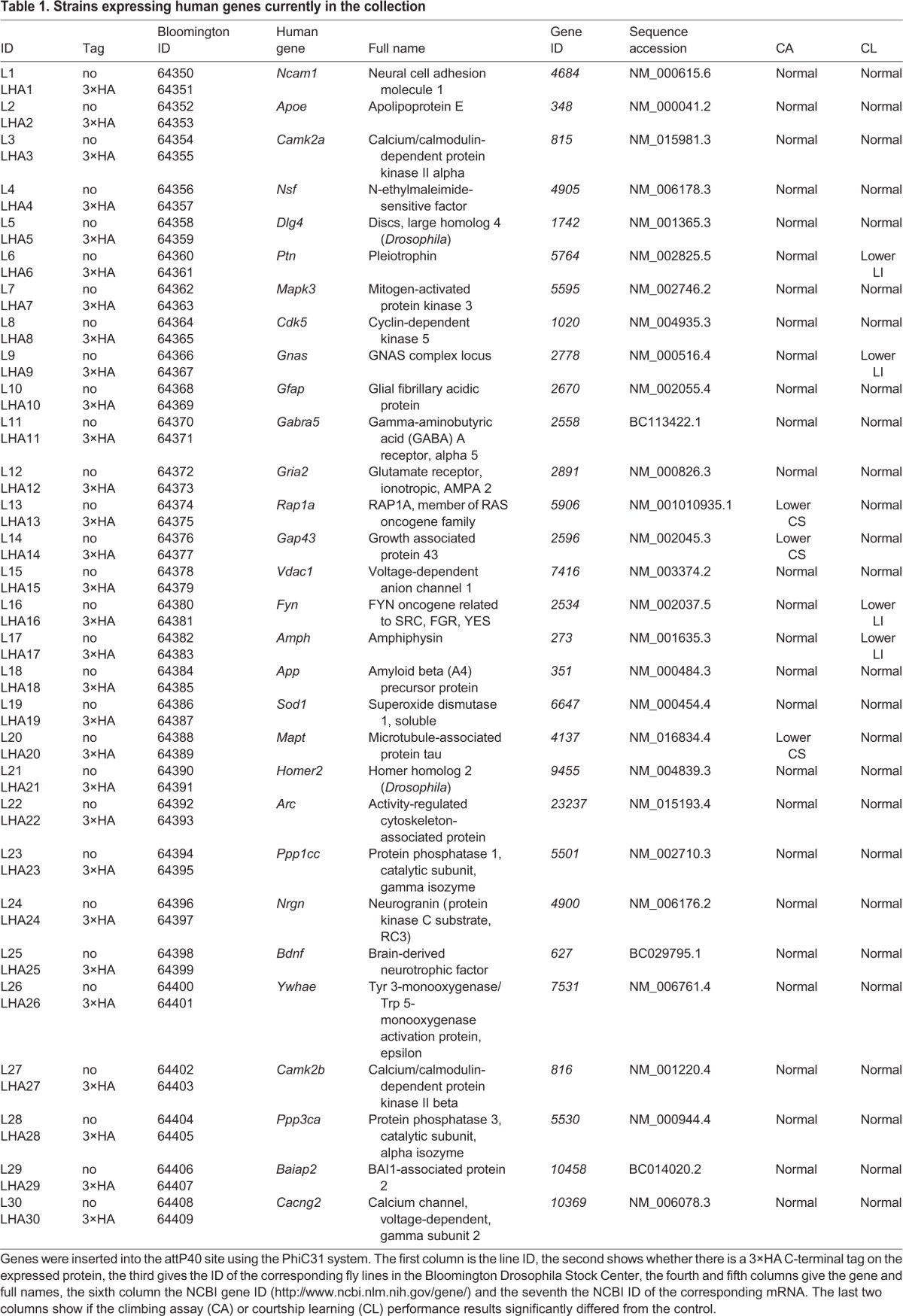
**Strains expressing human genes currently in the collection**

Using an optimised combination of gene synthesis where necessary, Gateway™ (Life Technologies) high throughput cloning and *Drosophila* PhiC31 transformation (Bischof et al., 2007; Markstein et al., 2008) we generated the resource, a collection of transgenic, ‘humanised’, fly strains expressing human synaptic genes under the control of UAS sequences. For every gene selected we made a fly strain expressing the native version as well as a strain expressing a C-terminal triple HA (3×HA) tagged version in order to maximise downstream application potential. The expression of the protein was verified with a western blot using an antibody against the HA tag and, where possible, the human protein.

### Phenotypic assay results

Each strain was visually checked for gross anatomical defects or severe behavioural defects (not shown). We then tested the collection using two common phenotypic assays. The first is a climbing (negative gravitaxis) assay to test for motor phenotypes through the loss of climbing ability and the second is a courtship learning assay for quantifying a more complex cognitive task. Each strain was crossed with a Gal4 driver appropriate for the assay. We used the pan-neuronal elav-Gal4 (Yao and White, 1994) for the climbing assay and the mushroom body (MB)-specific 30y-Gal4 for the courtship learning assay, since the MB is associated with courtship learning in *Drosophila* (McBride et al., 1999). We then assayed the first generation progeny of these crosses. Note that the type of line (3×HA tagged or non-tagged) tested was the one we had confirmed the transgene's expression for and in the majority of cases that was the line expressing the 3×HA tagged version of the construct.

**Fig. 1.**
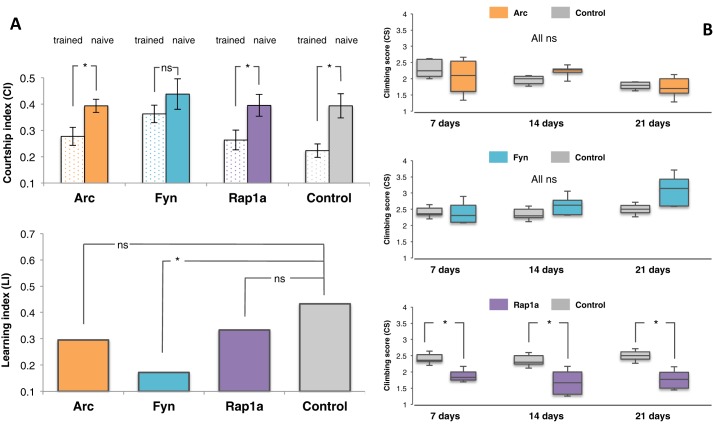
**Results of the courtship learning and climbing assays, quantifying learning and memory and motor phenotypes, respectively, for selected strains in the collection.** (A) Results of the courtship learning assay for the selected genotypes 30y-Gal4; UAS-Arc;+(Arc; *N*_trained_=14, *N*_naive_=23), 30y-Gal4; UAS-Fyn;+(Fyn; *N*_trained_=17, *N*_naive_=19), 30y-Gal4; UAS-Rap1;+(Rap1a; *N*_trained_=28, *N*_naive_=27) and the respective driver and wild type cross 30y-Gal4;+;+ (Control; *N*_trained_=23, *N*_naive_=24). All strains exhibit courtship within a relatively normal range (not shown). All lines with the exception of Fyn show significant differences between the Courtship Index (CI) of the trained (empty bar) and naive (filled bar). This translates to the Fyn expressing flies having a significantly lower Learning Index (LI) compared to controls. ANOVA showed that the CI difference between trained and naïve flies for Arc, Rap1a and control were significant (see Table S1 for *P*-values) while the CI difference between trained and naïve flies for Fyn was not. Multiple testing showed that the LIs Fyn expressing flies were significantly lower than the control (*P=0.0359). Error bars show s.e.m. (B) Results of the climbing assay for the following genotypes: elav-Gal4; UAS-Arc;+(Arc), elav-Gal4; UAS-Fyn;+(Fyn), elav-Gal4; UAS-Rap1;+(Rap1a) and the respective driver and wild type cross elav-Gal4;+;+ (Control). *N*=50 (5 replicate vials of 10 flies each). For significance testing: ns, *P*>0.05; *P<0.05. Arc and Fyn show similar climbing abilities to the Control, whereas the Rap1a-expressing line shows a significant difference in the climbing score compared to the control. *P*-values are 0.0019, 0.0146 and 0.0210 for 7, 14 and 21 days, respectively (see also Table S2). Note how the Arc-expressing line in the figure, like most of the other strains (not shown) has no significant phenotype in the phenotypic assays.

Of the humanised lines tested, four showed a cognitive phenotype (lines expressing Fyn, Amph, Gnas and Ptn, all tested in the 3×HA tagged version line) and three showed a motor phenotype (lines expressing Rap1a, Gap43 and Mapt, all tested in the 3×HA tagged version line). Details are provided in Tables S1 and S2, respectively. Fig. 1 shows example results for the strains expressing Arc, Fyn and Rap1a. Of these genes, Arc has no known fruit fly ortholog, while Fyn and Rap1a have one-to-many ortholog relations with the Src64B and Roughened genes respectively. In mammals, all three genes have been implicated in plasticity and behaviour (Plath et al., 2006; Kojima et al., 1997; Morozov et al., 2003; Grant et al., 1992).

These results highlight three distinct potential outcomes of the search for gain-of-function phenotypes in the collection. Expression of Arc does not introduce any motor or cognitive phenotypes measurable with the assays used. An interpretation of the latter could be due to the lack of orthologues and by extension other interacting protein partners in the cellular context of *Drosophila*. Fyn and Rap1a introduce specific cognitive and motor gain-of-function phenotypes respectively.

## DISCUSSION

We have produced a collection of fruit fly strains and made it available to the research community. The fruit fly lines in this resource allow the study of the function of human synaptic genes in the context of the *Drosophila* central nervous system. As an example of this type of application we tested strains in the collection and found gain-of-function phenotypes with a frequency ranging between 10% (motor) and 13.3% (cognitive). Interpretation of the gain-of-function phenotypes could help with the functional characterisation of these genes, starting from the *Drosophila* context. For those lacking a phenotype they present platforms upon which the effect of gene variants may be studied in the fly.

Other potential applications could extend to attempting to modulate the extent of these, or other, phenotypes by using small drug-like molecules. The latter is particularly useful for testing the effect of new candidate drugs on a human target or its allelic variants. Beyond the quantification and attempts to pharmaceutical modulation of phenotypes there are also other applications such as the proteomic identification of immunoprecipitated samples, in order to discover native proteins that interact with the trans-protein. This could be particularly useful in the case of strains used as disease models and the HA tagged versions could facilitate this.

Finally the design of the collection allows the study of binary interactions though genetic combination and screening. Based on the PhiC31 integrase insertion system we have used, in order to generate combinatorial models all one has to do is use two transgenic strains, one with a construct inserted in one landing site (e.g. attP40 on chromosome 2), including the lines made available here, and a second in another site (e.g. attP2 on chromosome 3), using the same process as here, along with simple genetic crosses. In addition to that, the wide availability of RNAi stocks (Dietzl et al., 2007) allows the silencing of the native ortholog genes, where these exist, for more refined downstream studies. The investigation of genetic interactions is very useful in order to dissect the mechanisms of actions in various disease models using an organism as versatile as *Drosophila*.

The fly strains described here are available from the Bloomington Stock Center at Indiana University (http://flystocks.bio.indiana.edu).

## MATERIALS AND METHODS

### Human synaptic gene selection

We compiled a list of synaptic genes from proteomics pull-down experiments or co-immunoprecipitation using synaptic baits and annotated it for disease associations, synaptic plasticity and behaviour associations. The list was sourced from the Genes2Cognition database publically available datasets (http://www.genes2cognition.org; Croning et al., 2009) as well as the SynSysNet dataset (von Eichborn et al., 2013). We prioritised genes on the basis of whether and how often they appear in the aforementioned lists, their synaptic specificity and whether they were associated to reported behaviours, plasticity processes or disease annotations. A closer inspection of the resulting dataset revealed that it was biased against some genes associated with neurodegenerative diseases so we added several of these.

### Fly stocks, husbandry and maintenance

The fly stocks were fed on standard cornmeal medium maintained at 19°C. Experimental crosses were performed and the F1 progeny was maintained on the same medium at 25°C.

### Plasmids, cloning and transformation

We used the Gateway^TM^ cloning system (Life Technologies) in order to maximise the efficiency of the pipeline. In addition to that we decided to transform the flies using the *Drosophila* PhiC31 system (Bischof et al., 2007; Markstein et al., 2008) with the attP40 insertion site stock. This allows us to (a) control for position effects in the expression of the gene and (b) in combination with a different landing site stock (e.g. attP2) leaves the potential for combinatorial models open. In order to satisfy the above combination we used the Gateway compatible plasmids by FlyC31 (http://www.flyc31.org/) (Bischof et al., 2007). These come in non-tagged (pUASg) and a 3×HA C-terminal tagged (pUASg-HA) versions. The sequences of the inserts were confirmed post-synthesis and also with a Polymerase Chain Reaction (PCR) and sequencing at the ‘Expression clone’ stage using forward and reverse primers recommended for the sequence of the FlyC31 plasmids (forward: CTGCAACTACTGAAATCTGCCAAG, reverse: CCGCATAGTCAGGAACATCG). Transformation of the attP40 insertion site stocks was done by Genetic Services Inc (Cambridge, USA).

### Western blot confirmation of expression

Fly heads were homogenized in RIPA buffer (Sigma-Aldrich, UK), centrifuged, and the supernatant was collected. Proteins were separated on SDS-PAGE gel and then transferred onto membrane using the iBlot^®^ Transfer Stack, nitrocellulose (Life Technologies). Blocking in 5% skimmed milk was followed by overnight primary antibody incubation. The primary antibodies used were anti-HA (Roche) as well as, where available, primary antibodies against the native human genes. The primary antibody concentration used was 1 μg/μl for the protein specific antibodies (anti-Ncam1, UMAB83, Origene and anti-Nsf, ab16681, Abcam) and a 1:1000 dilution for the mouse anti-HA. Membranes were incubated with the secondary antibody (GE Healthcare) at a 1:5000 concentration for one hour, followed by signal detection using the Amersham ECL system (GE Healthcare). See Fig. S1 for a summary of the results.

### Climbing assay

The climbing assay was performed with progeny of a c155 (elav-Gal4) cross, expressing the transgene pan-neuronally. Flies were tested at the same time of day to exclude circadian rhythm effects. Ten replicate transparent 40-cm long rigid tubes, containing 10 flies each, were simultaneously dropped on bench from the same height using an in-house designed apparatus. The whole process was recorded on High Definition video. Using the video we identify the frame of the moment of impact and extract the frame located 15 seconds later. The vials in the frames are then separated in four equal segments and the number of flies in each segment was counted. For each replicate vial a climbing score (CS) is calculated as a weighted average of these segment counts, with weights assigned as w_s_=s (where segment s=1, 2…). This weighting represents rewarding the innate climbing behaviour. The climbing score is averaged for groups of replicate vials. The groups of replicate CS values we compared for significant differences using one-way ANOVA. The significance cut-off was a *P*-value <0.05.

### Courtship learning assay

Courtship behaviour of the fruit flies is a vigorous pursuit of the male after a female stimulated by the female pheromones (Tompkins and Hall, 1981). As the behaviour is innate, even the virgin males are able to perform. However, a virgin male courting a previously mated female is usually rejected and learns to suppress further courtship towards other females (Tompkins et al., 1983). Thus testing the courtship behaviour tests learning and memory abilities as well. The protocol for courtship conditioning started with leaving 7-day-old virgin males for 30 min in the courting chamber without the female (rest phase). Next the mated female was introduced into the chamber with the male for 30 min (learning phase). After that time, male and female were separated by the divider for another 30 min (rest phase) and then the male associative memory was tested for 6 min. The test phase was recorded with the use of a digital camera in 23-25°C and 30-32% humidity. Scoring was done blind and performed with the use of FlyAnnotate v1.0 (developed by James Heward, Actual Analytics Ltd at time of development). The various behaviours connected to courting like orientation, pursuit, wing vibration, genital licking and attempted copulation (O'Dell, 2003) were scored as courting by pressing a relevant key on the keyboard. The courtship index (CI) was calculated by dividing the number of frames (time) scored as courtship by the total number of frames (time) of the movie (Tompkins et al., 1983; O'Dell, 2003). Scores of 0 were discarded as invalid. Having the CI of trained and naïve flies, it was possible to calculate the learning (performance) index (LI) (Kamyshev et al., 1999): LI=((CI_naïve_−CI_trained_)/CI_naïve_)×100. ANOVA was performed to check whether the CI of both trained and naïve was significantly different. Multiple testing was performed to see if the LI of the trained and the naïve flies was significantly different (Kamyshev et al., 1999). The significance cut-off was a *P*-value <0.05.
